# Enrichment of Hamburger Meatballs With Psyllium: Effects on Postprandial Lipidemia, Glycemia, Appetite, and Food Intake in a Triple‐Blind Randomized Controlled Crossover Trial

**DOI:** 10.1002/fsn3.71066

**Published:** 2025-10-09

**Authors:** Ahmet Murat Günal, Hande Öngün Yılmaz, Murat Baş

**Affiliations:** ^1^ Department of Nutrition and Dietetics Faculty of Health Sciences, Haliç University Istanbul Türkiye; ^2^ Department of Nutrition and Dietetics Faculty of Health Sciences, Bandırma Onyedi Eylül University Balıkesir Türkiye; ^3^ Department of Nutrition and Dietetics Faculty of Health Sciences, Acıbadem Mehmet Ali Aydınlar University İstanbul Türkiye

**Keywords:** appetite, fast food, food intake, glycemia, hamburger, postprandial lipidemia, psyllium, soluble fiber

## Abstract

Psyllium may improve the nutritional profile of fast foods. This study enriched hamburger meatballs (HM)—a popular low‐fiber fast food—with psyllium, evaluated their sensory acceptability, and examined effects on postprandial lipidemia, glycemia, food intake, hunger, and satiety. HM containing 5% and 7.5% psyllium was first tested in a triple‐blind sensory panel of 12 trained dietitians; no significant differences in preference or hedonic scores (*p* > 0.05) supported 7.5% as suitable for further trials. A randomized, triple‐blind, crossover trial with 25 healthy adults then compared psyllium‐enriched HM (PEHM) to control HM (CHM). Hunger and satiety ratings were recorded hourly for 6 h; fasting and 2‐h postprandial blood samples; and prospective food intake records were collected. Data were analyzed in SPSS 22.0 using parametric and nonparametric tests, post hoc comparisons, and repeated measures mixed ANOVA. PEHM, containing 7.5% psyllium, was well‐tolerated and compared to CHM, it significantly reduced the postprandial increase of participants' triglyceride (*p* = 0.015), VLDL (*p* = 0.015), and glucose levels (*p* = 0.036). PEHM consumption also led to lower prospective energy (*p* = 0.009), total fat (*p* = 0.016), PUFA (*p* = 0.005), and omega‐6 intake (*p* = 0.006) compared to control. Hunger scores were significantly lower at the 4th (*p* = 0.013) and 5th (*p* = 0.003) post‐meal hours, while satiety scores were significantly higher at the 3rd (*p* = 0.025), 4th (*p* = 0.013), and 5th (*p* = 0.029) hours compared to CHM. These findings suggest that psyllium enrichment of fast foods can help reduce risk factors for chronic diseases without compromising sensory quality. Turkish Patent and Trademark Office registered the invention with a utility model certificate number TR2023003533Y.

**Trial Registration:** Clinicaltrials.Gov: NCT05825963

## Graphical abstract

The two‐block randomized controlled triple‐blind sensory analysis panel showed sensory and hedonic properties of 7.5% psyllium‐enriched hamburger meatballs (PEHM) were similar to control. Randomized controlled triple‐blind crossover trial results suggest that compared to control, PEHM significantly reduced postprandial triglyceride, VLDL, and glucose levels, alongside prospective energy, total fat, PUFA, and omega‐6 intake. Hunger decreased at the 4th and 5th hours; satiety increased at the 3rd, 4th, and 5th hours.

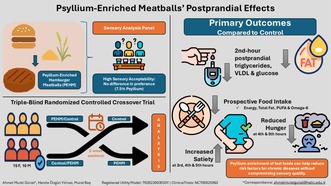

## 1 Introduction

Psyllium seed husk, derived from the *Plantago ovata* plant, stands out for its high soluble dietary fiber content. Psyllium can absorb up to 80 times its weight in water. This high hydrophilic property and the ability to form mucilage make it particularly useful in the food industry as a thickening and gelling agent (Phan et al. 2020). This mucilage does not ferment in the intestine and leaves the gastrointestinal system unchanged (Fischer et al. 2004).

The viscous gel formed by psyllium increases the viscosity of chyme, delaying the interaction of complex carbohydrates with digestive enzymes and slowing digestion and glucose absorption, reducing the peak level of postprandial glucose concentration in the blood (Gibb et al. 2015). This delay stimulates the receptors in the distal ileum, causing the release of glucagon‐like peptide‐1 (GLP‐1), which has effects such as reducing appetite and increasing glucose‐dependent insulin secretion (McRorie Jr. 2015). As a result of increased viscosity, bile cannot be absorbed effectively and is excreted in the feces, causing a decrease in the bile acid pool, stimulating hepatocytes to produce LDL cholesterol receptors and increase their numbers. This increases the uptake of LDL from the blood and thus lowers LDL and total cholesterol concentrations without affecting HDL cholesterol levels (Narayanan and Pitchumoni 2020). The United States Food and Drug Administration (FDA) has reported that adding over 7 g of psyllium to a diet low in saturated fat and cholesterol may reduce the risk of coronary heart disease (FDA 1998). When consumed with or before a meal, psyllium helps to stimulate the stretch receptors in the stomach by taking up space, delaying gastric emptying, and allowing food to reach the distal regions of the small intestine, contributing to a feeling of prolonged satiety. Also, psyllium may play a role in reducing postprandial glycemic peaks and lowering insulin requirements, leading to this feeling (Wanders et al. 2011). Due to its effects on digestion and appetite, consuming psyllium may reduce daily energy intake and contribute to weight loss (Howarth et al. 2001).

The American National Institutes of Health, Food and Nutrition Board, recommends that adult women consume 25 g of fiber per day and 38 g for men as the daily reference intake. As a practical calculation method, consuming 14 g of fiber per 1000 cal is also recommended (Trumbo et al. 2002). According to the latest Türkiye Nutrition and Health Study, average fiber consumption is 20.3 g/day for women and 24.4 g/day for men (TR Ministry of Health 2019). Moreover, the Türkiye Nutrition Guide gives fiber a place in the “foods and nutrients that need to be increased in consumption” section to highlight its importance (TR Ministry of Health 2022).

Today, people consume large quantities of fast‐food products, which are poor in vegetables and fruit groups and high in processed refined grains, leading to a lower fiber content (Isganaitis and Lustig 2005). Fast food is particularly popular among young adults. A 2019 survey conducted in France reported that 84% of respondents aged 18 to 24 expressed a preference for fast‐food restaurants (OpinionWay 2019). Similarly, a recent survey in Italy found that among individuals aged 18 to 34, 28% consumed fast food once a week, while 27% did so twice a month. In contrast, these rates declined to 14% and 15%, respectively, among respondents aged 35 to 54 (U.Di.Con 2024).

Hamburgers are one of the most consumed fast‐food products worldwide. It was the second most ordered fast food item with a 25% rate after pizza (29%) in the US (InMobi 2023). As of the fourth quarter of 2024, french fries, fried chicken, and hamburgers were the most popular American dishes in the United States. An average of around 84% of respondents had a positive opinion of hamburgers (YouGov 2025).

While the physiological benefits of psyllium are extensively studied, its integration into popular low‐fiber foods like hamburgers represents a novel intervention strategy. This approach not only addresses fiber deficiency in modern diets but also innovatively bridges the gap between convenience foods and health promoting nutrition. In this study, we aimed to enrich the hamburger meatball with psyllium without impairing its sensory properties and to investigate the effects on acute postprandial lipemia and glycemia, prospective food intake, and some appetite indicators. The main hypotheses of the study are:

– There is no difference in sensory analysis results between hamburger meatballs enriched with psyllium and those that are not enriched.
– The rise in postprandial lipids after consuming psyllium‐enriched hamburger meatballs is lower than that of classic hamburgers.
– The rise in postprandial glycemia after consuming psyllium‐enriched hamburger meatballs is lower than that of classic hamburgers.
– The feeling of satiety lasts longer, and the feeling of hunger increases more slowly after consuming psyllium‐enriched hamburger meatballs compared to classic hamburgers.
– Daily food intake after consuming psyllium‐enriched hamburger meatballs is less than that of classic hamburgers.

## 2 Materials and Methods

The study has two parts. First, it involves enriching hamburger meatballs (HM) with psyllium and conducting a two‐block randomized controlled triple‐blind sensory analysis panel to assess sensory and hedonic appreciation. Second, it involves conducting a randomized controlled triple‐blind crossover trial to assess its effectiveness on postprandial biochemical markers, subjective appetite, and prospective food intakes.

The study adhered to the guidelines outlined in the Declaration of Helsinki and received approval from the Acıbadem Mehmet Ali Aydınlar University Medical Research Evaluation Board's (ATADEK) ethics committee (2021‐24/31). Participation in the study is based on voluntariness. Individuals participating in the sensory analysis panel and experimental period were informed and given written informed consent before the research.

Turkish Patent and Trademark Office (Türk Patent) deemed psyllium‐enriched hamburger meatballs as a utility model on 21 March 2024 with certificate number TR 2023 003533 Y.

### 2.1 Participants

The sensory analysis panel, which formed the first part of the study, was conducted with 12 trained panelists, all of whom were dietitians. Panelists were aged between 22 and 31, did not smoke, and did not have any illnesses that could affect their sense of taste or smell on the panel day. The panelists were informed about the study and reminded of the points to consider during the sensory analysis.

To determine the appropriate sample size for the experimental period, an a priori power analysis was conducted using the G*Power 3.1 software package. The primary outcome used for this power analysis was serum triglyceride levels, and the statistical analysis was based on an *F* test—ANOVA: Repeated measures, within–between interaction, reflecting the hypothesis that there would be a significant interaction between time (pre‐ and post‐intervention) and condition (psyllium‐enriched vs. control HM) on triglyceride concentrations. Based on a similar intervention study (Suter et al. 2005), the effect size was calculated as 0.9826827. Using this effect size, the required sample size was determined for a power of 0.80 and an *α* level of 0.05, resulting in a minimum of 36 measurements. In the context of this crossover design, where each participant provides data for both intervention periods, this corresponds to a required sample size of 18 participants. Ultimately, 25 participants completed the trial. A post hoc power analysis was subsequently performed based on the observed changes in serum triglyceride levels to confirm whether the final sample size provided sufficient power. The observed effect size was 0.8241634, and the resulting power was calculated to be 0.8169755, confirming that the study retained adequate power with 25 participants.

The inclusion criteria for the study were voluntary participation, aged between 19 and 35 years due to high fast‐food consumption (OpinionWay 2019; Singh et al. 2021; U.Di.Con 2024) and a body mass index (BMI) between 18.5 and 25 kg/m^2^. High fast‐food consumption was defined as consuming fast food at least once a week, and only individuals meeting this criterion were included in the study. The exclusion criteria were initially solely based on self‐reported medical history and included having any chronic or metabolic disorders, any known food allergies, and the use of lipid‐lowering agents. During the trial, two participants were excluded due to the detection of elevated LDL levels.

**FIGURE 1:**
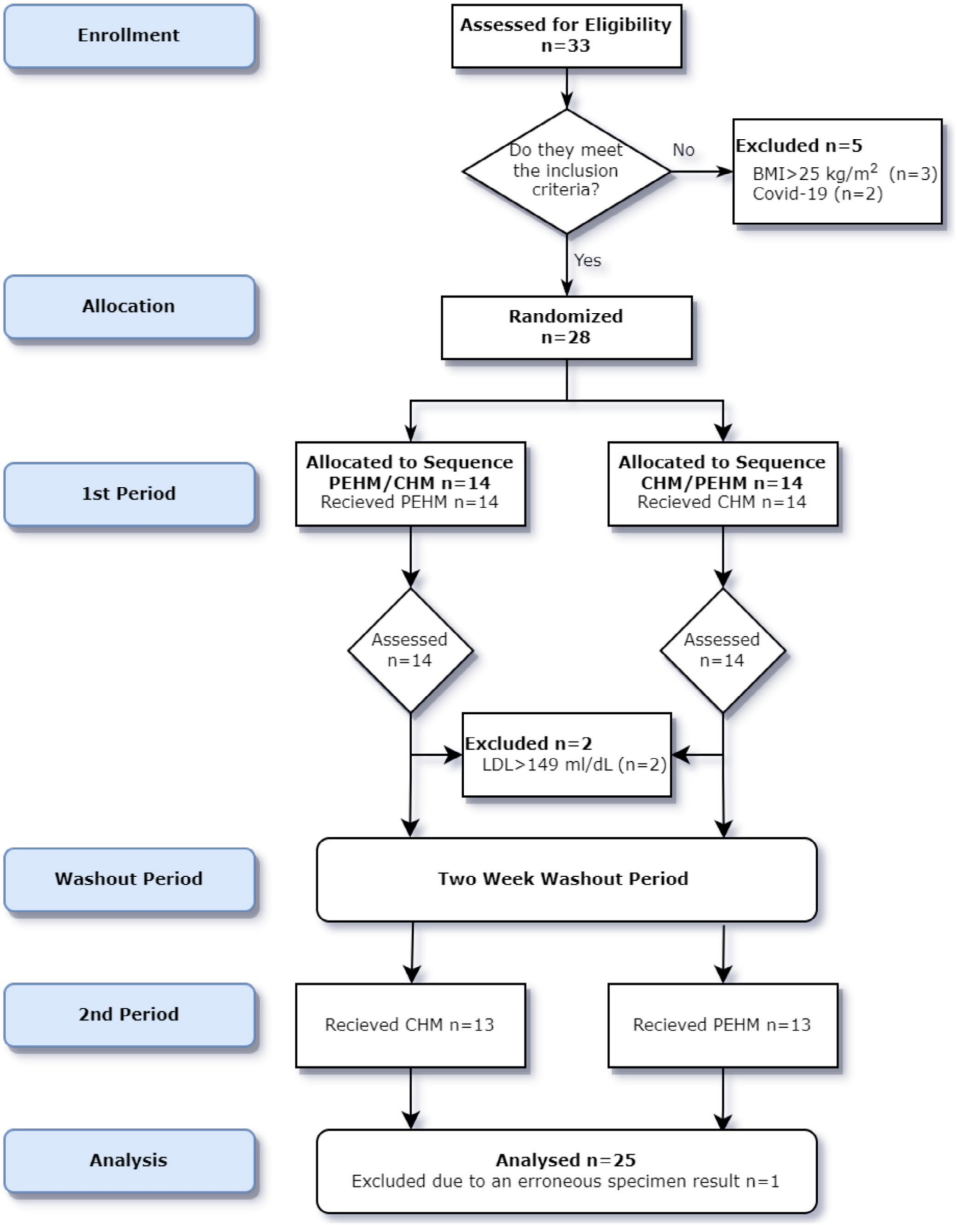
CONSORT flow diagram for crossover trial.

A total of 33 individuals who accepted the invitation made by the researcher through social media platforms were evaluated. Since the announcement specifically targeted individuals who consumed fast food at least once a week, all respondents met this criterion. Three participants were not included in the study due to a BMI above 25 kg/m^2^, two were unable to participate due to being in COVID‐19 quarantine on experiment days. During the study, three participants were excluded due to dyslipidemia (*n* = 2) and an error in their laboratory results (*n* = 1). The remaining 25 participants (15 female, 10 male) constituted the sample for the study (Figure 1). In line with the results, a post hoc power analysis was performed again to ensure whether the sample number was sufficient, and the effect size was found to be 0.8241634. It was concluded that a sufficient sample size was reached with 25 people included.

### 2.2 Enrichment of HM and Sensory Analysis Panel

A mixture of 20% fat ground meat, 1.6% salt, and 0.4% black pepper was used to prepare the meatball dough. The ground meat was divided into three equal parts, and organic psyllium powder was added to two of them at a rate of 5% and 7.5%, while no intervention was made to the third part (Control). An independent academic randomly assigned six 3‐digit numbers using the Google Random Number Generator to ensure that the panel was triple‐blind.

The sensory analysis panel was conducted in two separate sessions (blocks) in a laboratory setting. In each block, samples were served to participants in a randomized order using a computer‐generated randomization list, in a triple‐blind design. To assess panelist reliability, the three meatball types (control, 5% enriched, and 7.5% enriched) were evaluated in two separate blocks. In each block, the three meatballs were assigned unique, three‐digit random codes, resulting in a total of six different codes being used across the two sessions (3 products × 2 blocks = 6 codes). This approach was designed to present the products as six distinct samples, enabling us to evaluate the consistency of the panelists' ratings for the same product across different sessions. The products were presented to the panelists one by one, and they were asked to rate six factors: the taste, smell, appearance, mouthfeel, juiciness, and texture on a scale of 1 to 9 (1: very bad, 9: very good), and the average overall preference was calculated. In addition, a five‐point Likert (1: definitely disliked, 5: definitely liked) hedonic scale was also used to measure general appreciation.

### 2.3 Experimental Period

According to panel results, the meatball enriched with 7.5% psyllium was selected for the experimental period.

Participants were asked to keep a record of their food intake for the previous 24 h, to avoid heavy physical activity, to abstain from alcohol before the first day of the study, and to arrive at the study in a state of fasting for 12 h. On the study day, the participants' sociodemographic information was collected, anthropometric measurements (height, weight, and fat percentage) were taken, and fasting blood samples were collected by a nurse. The participants then consumed each hamburger on different days, with or without (control) 12 g of psyllium, each containing 85 g of hamburger bread, 160 g of 20% fat beef, 2.56 g of salt, and 1.28 g of black pepper, and were asked to consume them with 200 mL of water. After waiting for 2 h in a designated room, postprandial blood samples were collected in the same order and sent for analysis.

The participants were invited back for the second intervention, following a 2‐week washout period.

### 2.4 Randomization, Allocation and Blinding

The PEHMs and CHMs were placed in identical storage containers and frozen at −18°C in a deep freezer. An independent academic used the Google Random Number Generator to randomly assign two 3‐digit numbers and coded each group accordingly to ensure that the experimental period was triple‐blind.

To eliminate any potential for researcher led allocation bias during the first intervention, a physical randomization method was employed. An equal number of hamburgers were prepared with PEHM and CHM (14 for each) and presented together on a single table. The three‐digit codes, concealed from both participants and researchers, were written under the plates. Each participant's free selection of one hamburger on the first trial day served as the randomization event, assigning them to their allocation sequence for the duration of the trial (e.g., a participant who chose a PEHM on day one was allocated to the PEHM–CHM sequence). The researcher recorded which participant consumed which coded hamburger. Following the 2‐week washout period, participants were provided with the alternate hamburger they had not consumed during the first period.

The guidelines outlined in the CONSORT 2010 statement (Schulz et al. 2010) and its extension to randomized crossover trials (Dwan et al. 2019) were followed during the conduct of the study.

### 2.5 Measurements

#### 2.5.1 Food Intake Records

A 24‐h dietary recall (National Institutes of Health (NIH) n.d.‐a) of participants was obtained before each intervention day to assess potential habitual dietary differences that might influence postprandial responses. Since participants arrived at each intervention day following a 12‐h overnight fast, their fiber intake on the previous day could potentially affect the metabolic outcomes measured postprandially. Therefore, the 24‐h recalls were collected to control for such acute dietary effects. Additionally, participants were asked to keep a prospective food record (National Institutes of Health (NIH) n.d.‐b) for the day following each intervention to monitor any subsequent changes in food intake. All dietary records were analyzed using the Turkish Nutrition Information System (BeBiS) program version 8.0 (Beslenme Bilgi Sistemi (BeBiS) n.d.). When analyzing the 24‐h dietary recalls of the participants before the trial days, it was found that the intakes of energy, macronutrients, and fiber were all similar (*p* > 0.05). The Supporting Information section includes the table presenting participants' baseline dietary intake findings prior to the intervention days.

#### 2.5.2 Subjective Hunger and Satiety Evaluations

Subjective evaluations of hunger and satiety were conducted using a 100 mm horizontal visual analog scale (VAS), with participants marking their perceived sensations on a line anchored with opposing descriptors (0: not at all, 100: extremely). The VAS is recognized for its sensitivity and reliability in capturing subtle fluctuations in appetite‐related sensations (Åberg et al. 2023; Parker et al. 2004). In the present study, these assessments were performed at baseline and subsequently at hourly intervals for 6 h on each of the two intervention days.

While the VAS can encompass multiple components, this study intentionally focused only on the core parameters of “hunger” and “satiety.” This focused approach was chosen for two main reasons. Firstly, it aligns directly with the study's primary objective of tracking core appetite sensations. Secondly, it served to reduce participant burden, a critical consideration for maintaining data quality and participant compliance during repeated hourly assessments. This streamlined methodology is further supported by literature indicating that hunger and satiety scores are highly correlated with other appetite metrics (e.g., fullness, desire to eat) and often provide comparable overall conclusions (Drapeau et al. 2005; Gibbons et al. 2019).

#### 2.5.3 Biochemical Measures and Calculations

Five milliliters of blood samples were collected twice from the antecubital vein using Vacusere CAT serum tubes with gel (Disera, Turkey). The first sample was obtained following a 12‐h overnight fast (baseline), and the second was collected at the second hour postprandially. This procedure was repeated on the second trial day, resulting in a total of four blood samples collected per participant over the course of the study. Samples were coded and centrifuged for 10 min at 4000 rpm using an NF 200 model centrifuge (NÜVE, Turkey) within 15 min of collection. Triglycerides, VLDL, LDL, HDL, glucose, and insulin values were determined using the chemiluminescent microparticle immunoassay (CLIA) method with Architect i1000 immunoturbidimetry and Architect ci4100 integrated immunochemistry analyzers (Abbott, USA). The total cholesterol levels of the participants were calculated using the Friedewald formula and the homeostasis model assessment of insulin resistance (HOMA‐IR) index; the value was calculated using the formula: [HOMA‐IR = glucose (mg/dL) × insulin (mU/L)/405] (Matthews et al. 1985).

### 2.6 Statistical Evaluation

All statistical analyses were conducted using SPSS version 22.0 (IBM Corp., Armonk, NY, USA). Descriptive statistics included percentages, means, standard deviations, and minimum and maximum values. The internal consistency reliability of the sensory evaluation scale—which assessed taste, smell, appearance, mouthfeel, juiciness, texture, and overall preference ratings—was evaluated using Cronbach's alpha. For bivariate analyses, independent samples *t*‐tests and paired samples *t*‐tests were used for normally distributed variables, while the Mann–Whitney U and Wilcoxon signed‐rank tests were applied for non‐normally distributed data. For comparisons involving more than two groups, one‐way ANOVA followed by Duncan's post hoc test or the Kruskal–Wallis H test with Dunn's post hoc comparisons were performed. To analyze repeated measures data, a Repeated Measures Mixed Way ANOVA was used, with the within‐subject factor being the experimental condition (psyllium‐enriched vs. control) and, when applicable, time. No between‐subjects factors were included due to the crossover design, in which each participant served as their own control. The assumption of sphericity was tested using Mauchly's test, and Greenhouse–Geisser corrections were applied where necessary. Post hoc comparisons were conducted using Duncan's test. Prior to inferential analyses, normality assumptions (assessed via skewness and Mardia's multivariate normality test), homogeneity of variances (Levene's test), and equality of covariance matrices (Box's *M* test) were evaluated. When appropriate, generalized linear models were also employed to control for potential covariates and minimize the risk of Type I error. All analyses were conducted at a 95% confidence level.

Guidance from Lo (2022) was considered during manuscript preparation to enhance clarity and coherence in reporting.

## 3 Results

### 3.1 Sensory Analysis Panel

In the first block of the sensory analysis panel, the control meatball received higher scores than the meatball enriched with 7.5% psyllium in terms of taste (*p* = 0.023). There were no differences found in the average ratings for other factors among the meatballs (*p* > 0.05). In the second block, all averages were found to be similar (*p* > 0.05).

Considering inter‐block comparison, participants gave higher scores for mouthfeel to the control meatball on the second evaluation (*p* = 0.018) when reevaluating the same meatball without knowing which one they had previously. No differences were found in the evaluations of the other meatballs and factors (*p* > 0.05). The internal consistency of the data was examined with Cronbach Alpha values and was found to be reliable.

Results of the in‐block comparison of different meatballs and inter‐block comparison of the same meatballs are given in Table 1.

**TABLE 1:** Comparison of in‐block and inter‐block sensory factors, overall preference, and hedonic appreciation of meatballs.

Measurement	1st block					2nd block					Inter‐block comparison					
	Control (*n* = 12)	5% (*n* = 12)	7.50% (*n* = 12)	*F*/*χ* ^2^	*p*_1_	Control (*n* = 12)	5% (*n* = 12)	7.50% (*n* = 12)	*F*/*χ* ^2^	*p*_2_	Control		5%		7.50%	
	*X̄* ± SD	*X̄* ± SD	*X̄* ± SD			*X̄* ± SD	*X̄* ± SD	*X̄* ± SD			*t*/*z*	*p*_3_	*t*/*z*	*p*_4_	*t*/*z*	*p*_5_
Taste^a^	6.50 ± 1.38	5.58 ± 1.16	5.17 ± 0.83	**4.228**_***F***_	**0.023** *	6.83 ± 1.11	5.67 ± 1.50	5.83 ± 1.27	4.228_*χ*_^2^	0.071	−0.886_*t*_	0.394	−0.085_*z*_	0.932	−1.526_*z*_	0.127
Smell	7.17 ± 1.40	6.58 ± 1.44	7.00 ± 1.60	0.967_*χ*_^2^	0.615	6.33 ± 1.67	6.67 ± 0.98	6.25 ± 1.22	0.334_*F*_	0.718	−1.556_*z*_	0.120	−0.192_*t*_	0.851	−0.777_*z*_	0.437
Appearance	7.25 ± 1.71	7.50 ± 1.24	7.17 ± 1.53	0.159_*F*_	0.854	7.25 ± 1.54	7.42 ± 1.16	6.17 ± 2.12	0.159_*χ*_^2^	0.208	−0.137_*z*_	0.891	−0.333_*z*_	0.739	−1.552_*z*_	0.121
Mouthfeel	6.08 ± 1.38	5.92 ± 1.51	5.58 ± 1.16	0.422_*F*_	0.659	7.08 ± 1.08	6.08 ± 1.44	5.83 ± 1.53	2.817_*F*_	0.074	**−2.360**_*z*_	**0.018** *	−0.297_*t*_	0.772	−0.442_*t*_	0.667
Juiciness	6.58 ± 1.88	6.67 ± 1.83	6.83 ± 1.27	0.069_*F*_	0.934	5.75 ± 1.60	6.42 ± 1.38	5.83 ± 1.99	0.069_*χ*_^2^	0.583	−1.481_*z*_	0.139	−0.142_*z*_	0.887	−1.706_*z*_	0.088
Texture	6.00 ± 1.41	6.33 ± 2.06	6.58 ± 1.44	0.370_*F*_	0.693	6.17 ± 1.85	6.25 ± 1.42	6.08 ± 1.56	0.032_*F*_	0.969	−0.632_*z*_	0.527	−0.121_*z*_	0.904	0.944_*t*_	0.365
Overall Preference	6.60 ± 0.99	6.43 ± 1.16	6.39 ± 0.77	0.150_*F*_	0.861	6.57 ± 1.10	6.42 ± 0.98	6.00 ± 1.25	0.838_*F*_	0.442	−0.257_*z*_	0.797	0.036_*t*_	0.972	−0.393_*z*_	0.694
Hedonic Appreciation	3.67 ± 0.99	3.17 ± 1.27	3.00 ± 0.86	1.312_*F*_	0.283	3.33 ± 0.89	3.83 ± 1.19	3.08 ± 1.24	1.400_*F*_	0.261	−0.518_*t*_	0.615	−0.378_*t*_	0.713	−0.171_*z*_	0.864
Cronbach *α*											0.875		0.820		0.613	

*Note:* Bold values indicate statistical significance at *p* < 0.05.

Abbreviations: *χ* ^2^: Kruskal Wallis *H* test statistic; *F*, Two‐Way Analysis of Variance; SD, standard deviation; *t*, dependent samples *t*‐test value; *X̄*, mean; *z*, Wilcoxon signed‐rank test.

^a^ Duncan post hoc, Control > 7.5% enriched.

* *p* < 0.05.

According to the sensory analysis panel results, it was deemed that the enrichment of meatballs with 7.5% psyllium is acceptable in terms of sensory factors, overall preference, and hedonic appreciation.

### 3.2 Experimental Period

This section presents the findings of the randomized controlled triple‐blind experimental period, which constituted the second part of the study.

#### 3.2.1 Descriptive Results of Participants

Participants who volunteered for the study were single, did not have any chronic illnesses or food allergies, and did not use any dietary supplements. One participant was pursuing a postgraduate degree, while the rest were at the undergraduate level. The average age was 21.12 ± 1.75 years. Anthropometric measurements of the participants are given in Table 2.

**TABLE 2:** Anthropometric measurements of the participants before the experiment (*X̄* ± SD).

Measurement	Female (*n* = 15)	Male (*n* = 10)	All (*n* = 25)	*t*	*p*
Height (cm)	163.73 ± 5.51	181.40 ± 8.06	170.80 ± 10.96	**−6.532**	**< 0.001** **
Weight (kg)	54.37 ± 6.46	78.41 ± 6.83	63.98 ± 13.65	**8.915**	**< 0.001** **
BMI (kg/m^2^)	20.35 ± 1.63	23.77 ± 0.94	21.71 ± 2.19	**−6.644**	**0.016** *
Muscle mass (kg)	40.50 ± 2.28	62.45 ± 4.98	49.28 ± 11.52	**−13.04**	**< 0.001** **
Fat mass (kg)	12.60 ± 4.60	14.00 ± 3.20	13.16 ± 4.08	−0.834	0.413
Fat (%)	21.18 ± 5.84	15.87 ± 3.26	19.06 ± 5.56	**2.606**	**0.008** **

*Note:* Bold values indicate statistical significance at *p* < 0.05 (*) and *p* < 0.01 (**).

Abbreviations: SD, standard deviation; *t*, dependent samples *t*‐test value; *X̄*, mean.

* *p* < 0.05.

** *p* < 0.01.

#### 3.2.2 Comparisons Between Pre‐Trial Days

When comparing the fasting blood levels of the participants on the trial days, it was observed that insulin levels were higher on the first trial day (*p* = 0.045), while the other parameters were similar (*p* > 0.05). Insulin levels were also found to be similar when females and males were compared separately (*p* > 0.05). When analyzing the 24‐h dietary recalls of the participants before the trial days, it was found that the intakes of energy, macronutrients, and fiber were all similar (*p* > 0.05). Detailed comparison of participants' fasting biochemical measurements on trial days and their energy and macronutrient intakes on pre‐trial days is given in the Supporting Information section (Tables S1 and S2).

#### 3.2.3 Experimental Results

In this section, the participants' changes in biochemical measurements, energy and macronutrient intakes, and subjective feelings of hunger and fullness during the experimental period were compared.

Both hamburgers resulted in a decrease in the participants' HDL and LDL levels, with no difference observed between the hamburgers (*p* > 0.05). When the PEHM was consumed, the increase in VLDL and triglyceride levels was statistically less (*p* = 0.015, both) and the decrease in glucose levels was greater (*p* = 0.036) compared to CHM. This effect was also seen in male participants only for VLDL and triglyceride levels (*p* = 0.047). The postprandial increase in insulin concentrations and HOMA‐IR values was also less when the PEHM was consumed compared to control, but this difference was not statistically significant (*p* > 0.05). A detailed comparison of participants' biochemical measurements according to the hamburgers consumed can be seen in Table 3.

**TABLE 3:** Comparison of participants' biochemical measurements according to the hamburgers consumed (*X̄* ± SD).

Measurement	Sex	PEHM					CHM					*F*	*p*_3_
		Fasting	Postprandial	Difference	*t*/*z*	*p*_1_	Fasting	Postprandial	Difference	*t*/*z*	*p*_2_		
Total cholesterol (mg/dL)	Female	170.23 ± 27.57	167.59 ± 28.7	−2.64 ± 6.17	1.658_*t*_	0.120	172.4 ± 22.77	172.33 ± 23.93	−0.07 ± 7.15	0.036_*t*_	0.972	0.136	0.715
	Male	166.36 ± 22.57	164.84 ± 21.53	−1.52 ± 6.91	0.696_*t*_	0.504	168.27 ± 24.39	166.45 ± 22.98	−1.82 ± 5	1.151_*t*_	0.280	0.030	0.864
	All	168.68 ± 25.26	166.49 ± 25.62	−2.19 ± 6.36	1.724_*t*_	0.098	170.75 ± 23.02	169.98 ± 23.25	−0.77 ± 6.32	0.608_*t*_	0.549	0.631	0.431
HDL (mg/dL)	Female	66.83 ± 13.13	63.95 ± 13.41	−2.89 ± 2.94	3.798_*t*_	**0.002** **	55.21 ± 9.36	53.16 ± 8.92	−2.05 ± 1.88	4.235_*t*_	**0.001** **	0.854	0.363
	Male	69.74 ± 11.32	66.41 ± 12.47	−3.33 ± 3.1	3.394_*t*_	**0.008** **	53.67 ± 7.44	50.83 ± 6.39	−2.84 ± 1.53	5.859_*t*_	**< 0.001** **	0.200	0.660
	All	68 ± 12.28	64.93 ± 12.83	−3.06 ± 2.95	5.189_*t*_	**< 0.001** **	54.6 ± 8.51	52.23 ± 7.94	−2.37 ± 1.76	6.732_*t*_	**< 0.001** **	1.026	0.316
LDL (mg/dL)	Female	88.97 ± 26.69	86.38 ± 26.29	−2.59 ± 3.69	2.723_*t*_	**0.016** *	98.51 ± 19.72	97.41 ± 20.37	−1.09 ± 5.4	0.784_*t*_	0.446	1.451	0.238
	Male	83.66 ± 22.01	82.75 ± 20.96	−0.91 ± 4.07	0.707_*t*_	0.498	95.22 ± 21.28	93.12 ± 20.34	−2.1 ± 2.46	2.695_*t*_	**0.025** *	0.625	0.439
	All	86.85 ± 24.58	84.93 ± 23.9	−1.92 ± 3.85	2.490_*t*_	**0.020** *	97.19 ± 19.99	95.7 ± 20.05	−1.5 ± 4.42	1.692_*t*_	0.104	2.843	0.098
VLDL (mg/dL)	Female	14.43 ± 4.71	17.27 ± 5.87	2.84 ± 2.66	−4.142_*t*_	**0.001** **	18.68 ± 9.7	21.76 ± 10.49	3.08 ± 3.35	−3.565_*t*_	**0.003** **	2.279	0.142
	Male	12.96 ± 3.73	15.68 ± 4.95	2.72 ± 3.46	−2.485_*t*_	**0.035** *	19.38 ± 9.98	22.5 ± 8.35	3.12 ± 4.29	−2.300_*t*_	**0.047** *	**4.553**	**0.047** *
	All	13.84 ± 4.32	16.63 ± 5.47	2.79 ± 2.93	−4.757_*t*_	**< 0.001** **	18.96 ± 9.61	22.06 ± 9.52	3.1 ± 3.67	−4.224_*t*_	**< 0.001** **	**6.304**	**0.015** *
Triglyceride (mg/dL)	Female	72.13 ± 23.54	86.33 ± 29.37	14.2 ± 13.28	−4.142_*t*_	**0.001** **	93.13 ± 47.85	108.8 ± 52.47	15.67 ± 16.34	−3.713_*t*_	**0.002** **	2.272	0.143
	Male	64.8 ± 18.63	78.4 ± 24.74	13.6 ± 17.31	−2.485_*t*_	**0.035** *	96.9 ± 49.89	112.5 ± 41.76	15.6 ± 21.45	−2.300_*t*_	**0.047** *	**4.553**	**0.047** *
	All	69.2 ± 21.6	83.16 ± 27.36	13.96 ± 14.67	−4.757_*t*_	**< 0.001** **	94.64 ± 47.67	110.28 ± 47.58	15.64 ± 18.12	−4.316_*t*_	**< 0.001** **	**6.302**	**0.015** *
Glucose (mg/dL)	Female	94.13 ± 6.01	86.27 ± 13.24	−7.87 ± 12.18	2.502_*t*_	**0.025** *	97.4 ± 7.01	91 ± 12.28	−6.4 ± 10.49	2.364_*t*_	**0.033** *	1.701	0.203
	Male	94.8 ± 4.29	79.8 ± 7.67	−15 ± 9.32	5.089_*t*_	**0.001** **	98 ± 8.15	94 ± 26.81	−4 ± 23.86	0.530_*t*_	0.609	2.833	0.110
	All	94.4 ± 5.3	83.68 ± 11.61	−10.72 ± 11.48	4.669_*t*_	**< 0.001** **	97.64 ± 7.33	92.2 ± 18.96	−5.44 ± 16.7	1.628_*t*_	0.116	**4.662**	**0.036** *
Insulin (μIU/mL)	Female	5.22 ± 1.51	18.88 ± 8.99	13.66 ± 8.74	−6.054_*t*_	**< 0.001** **	6.4 ± 2.28	21.17 ± 11.99	14.77 ± 11.54	−4.956_*t*_	**< 0.001** **	0.088	0.769
	Male	5.85 ± 2.12	24.57 ± 10.76	18.72 ± 10	−5.917_*t*_	**< 0.001** **	7.5 ± 3.02	25.83 ± 16.44	18.33 ± 15.17	−3.822_*t*_	**0.004** **	0.005	0.947
	All	5.47 ± 1.77	21.16 ± 9.94	15.68 ± 9.41	−4.373_*z*_	**< 0.001** **	6.84 ± 2.6	23.03 ± 13.81	16.19 ± 12.93	−6.263_*t*_	**< 0.001** **	0.767	0.385
HOMA‐IR	Female	1.22 ± 0.39	4.11 ± 2.19	2.89 ± 2.13	−5.275_*t*_	**< 0.001** **	1.56 ± 0.64	4.93 ± 3.23	3.37 ± 3.13	−4.178_*t*_	**0.001** **	0.241	0.627
	Male	1.38 ± 0.52	4.95 ± 2.52	3.57 ± 2.35	−2.803_*z*_	**0.005** **	1.83 ± 0.79	6.84 ± 6.41	5.01 ± 6.14	−2.803_*z*_	**0.005** **	1.034	0.323
	All	1.28 ± 0.44	4.45 ± 2.31	3.17 ± 2.2	−7.208_*t*_	**< 0.001** **	1.67 ± 0.7	5.7 ± 4.73	4.03 ± 4.53	−4.265_*t*_	**< 0.001** **	2.123	0.152

*Note:* Bold values indicate statistical significance at *p* < 0.05 (*) and *p* < 0.01 (**).

Abbreviations: *F*, repeated measures mixed‐way analysis of variance; *p*_1_, *p*‐value for the paired comparison between fasting and postprandial values within the PEHM condition; *p*_2_, *p*‐value for the paired comparison between fasting and postprandial values within the CHM condition; *p*_3_, *p*‐value for the comparison of the difference (postprandial—fasting) between the PEHM and CHM conditions (repeated measures mixed‐way ANOVA interaction effect).; SD, standard deviation; *t*, dependent samples *t*‐test value; *X̄*, mean; *z*, Wilcoxon signed‐rank test.

* *p* < 0.05.

** *p* < 0.01.

According to participants' food intake analyses results given in Table 4, when PEHM was eaten, compared to CHM, males, females, and together received less energy (*p* < 0.001, *p* = 0.047, *p* = 0.009, respectively) and more fiber (*p* < 0.001, *p* = 0.007, *p* = 0.001, respectively). In females, carbohydrate, protein, total fat, PUFA, and omega‐6 intake decreased (*p* = 0.010, *p* = 0.003, *p* < 0.001, *p* = 0.022, *p* = 0.024, respectively). When all participants were evaluated together, total fat, polyunsaturated fatty acid, and omega‐6 intake also decreased (*p* = 0.016, *p* = 0.005, *p* = 0.006, respectively). The increase in saturated fat intake was statistically significant when CHM was eaten, while this increase was not found to be statistically significant when PEHM was eaten, although there was no statistical difference between the hamburgers (*p* > 0.05).

**TABLE 4:** Comparison of participants' energy and macronutrient intake according to the hamburgers consumed (*X̄* ± SD).

	Sex	PEHM					CHM					*F*	*p*_3_
		Pre‐intervention	Post‐ intervention	Difference	*t*/*z*	*p*_1_	Pre‐intervention	Post‐ intervention	Difference	*t*/*z*	*p*_2_		
Energy (kcal)	Female	1236.01 ± 304.11	1240.59 ± 280.52	4.58 ± 496.23	−0.178_*z*_	0.859	1457.54 ± 367.59	1814.86 ± 365.38	357.32 ± 646.13	−2.563_*t*_	0.050	**44.006**	**< 0.001** **
	Male	2110.35 ± 614.75	1952.85 ± 339.44	−157.5 ± 767.91	−0.010_*t*_	0.993	1730.71 ± 597.73	2180.69 ± 478	449.99 ± 469.39	−2.330_*t*_	0.067	**4.556**	**0.047** *
	All	1585.75 ± 621.92	1525.49 ± 464.69	−60.25 ± 609.38	−0.044_*t*_	0.966	1566.81 ± 481.1	1961.19 ± 443.86	394.38 ± 572.97	−3.386_*t*_	**0.006** **	**7.385**	**0.009** **
Carbohydrate (g)	Female	140.54 ± 56.42	110.19 ± 42.91	−30.35 ± 81.05	2.524_*t*_	**0.036** *	155.13 ± 52.35	156.69 ± 52.52	1.56 ± 84.84	−0.655_*t*_	0.541	**7.692**	**0.010** *
	Male	206.52 ± 76.16	178.95 ± 67.09	−27.56 ± 85.14	−0.720_*t*_	0.523	158.60 ± 70.72	192.58 ± 67.62	33.98 ± 77.81	−0.941_*t*_	0.390	2.847	0.109
	All	166.93 ± 71.56	137.70 ± 62.80	−29.24 ± 80.95	0.959_*t*_	0.357	156.52 ± 58.96	171.04 ± 60.38	14.52 ± 82.05	−1.165_*t*_	0.269	3.604	0.064
Protein (g)	Female	47.66 ± 15.65	58.39 ± 15.17	10.73 ± 24.76	−0.780_*t*_	0.458	55.60 ± 16.51	77.58 ± 19.99	21.98 ± 25.79	−2.074_*t*_	0.093	**10.865**	**0.003** **
	Male	102.79 ± 29.90	98.14 ± 19.89	−4.65 ± 26.53	1.331_*t*_	0.275	88.10 ± 36.94	104.55 ± 26.22	16.45 ± 38.41	−2.125_*t*_	0.087	2.043	0.170
	All	69.71 ± 35.18	74.29 ± 26.03	4.58 ± 26.09	−0.129_*t*_	0.899	68.60 ± 30.57	88.37 ± 25.94	19.77 ± 30.81	−3.096_*t*_	**0.010** *	3.540	0.066
Total Fat (g)	Female	52.07 ± 16	61.06 ± 12.92	8.99 ± 18.51	−1.535_*t*_	0.163	66.61 ± 20.99	88.09 ± 17.83	21.48 ± 22.33	−2.452_*t*_	0.058	**17.039**	**< 0.001** **
	Male	93.95 ± 37.18	91.14 ± 16.32	−2.81 ± 47.96	1.247_*t*_	0.301	80.83 ± 24.47	107.69 ± 24.6	26.85 ± 17.52	−2.201_*t*_	**0.028** *	3.375	0.083
	All	68.83 ± 33.26	73.09 ± 20.58	4.27 ± 33.12	−0.969_*t*_	0.351	72.30 ± 23.07	95.93 ± 22.55	23.63 ± 20.33	−4.461_*t*_	**0.001** **	**6.204**	**0.016** **
SFA (g)	Female	20.40 ± 8.14	24.20 ± 4.60	3.80 ± 9.65	−1.137_*t*_	0.289	28.29 ± 8.78	33.69 ± 8.04	5.40 ± 10.73	−2.703_*t*_	**0.043** *	0.186	0.669
	Male	33.85 ± 14.80	35.34 ± 7.88	1.49 ± 18.03	−0.163_*t*_	0.881	27.07 ± 7.59	39.14 ± 11.03	12.07 ± 11.41	−3.122_*t*_	**0.026** *	2.460	0.134
	All	25.78 ± 12.88	28.65 ± 8.17	2.88 ± 13.32	−1.117_*t*_	0.286	27.80 ± 8.18	35.87 ± 9.53	8.07 ± 11.27	−4.174_*t*_	**0.002** **	2.218	0.143
MUFA (g)	Female	18.86 ± 7.45	23.23 ± 4.60	4.37 ± 7.95	−2.750_*t*_	**0.025** *	23.64 ± 9.78	32.02 ± 5.98	8.39 ± 9.73	−2.266_*t*_	0.073	1.532	0.226
	Male	33.28 ± 11.24	35.37 ± 8.01	2.09 ± 17.36	−0.730_*z*_	0.465	29.77 ± 11.91	38.60 ± 7.63	8.83 ± 7.60	−2.047_*t*_	0.096	1.267	0.275
	All	24.63 ± 11.47	28.09 ± 8.56	3.46 ± 12.30	−1.680_*t*_	0.119	26.09 ± 10.88	34.65 ± 7.31	8.57 ± 8.77	−3.195_*t*_	**0.009** **	2.859	0.097
PUFA (g)	Female	10.08 ± 5.21	7.20 ± 6.13	−2.88 ± 6.02	1.427_*t*_	0.191	10.22 ± 5.86	14.21 ± 7.13	3.99 ± 9.13	−0.455_*t*_	0.668	**5.923**	**0.022** *
	Male	21.02 ± 14.08	12.26 ± 5.84	−8.76 ± 13.9	1.988_*t*_	0.141	18.74 ± 9.38	20.63 ± 10.49	1.89 ± 11.50	−1.572_*t*_	0.116	3.484	0.078
	All	14.46 ± 10.96	9.23 ± 6.41	−5.23 ± 10.11	2.333_*t*_	**0.038** *	13.63 ± 8.43	16.78 ± 9.01	3.15 ± 9.97	−1.518_*t*_	0.157	**8.716**	**0.005** **
Cholesterol (mg)	Female	219.36 ± 187.93	181.75 ± 63.34	−37.61 ± 201.31	−0.770_*z*_	0.441	265.42 ± 157.85	282.19 ± 106.95	16.77 ± 136.89	−0.524_*z*_	0.600	0.749	0.394
	Male	582.21 ± 292.66	376.72 ± 57.73	−205.49 ± 274.28	−1.826_*z*_	0.068	639.98 ± 416.42	419.25 ± 223.16	−220.74 ± 434.58	−0.105_*z*_	0.917	0.009	0.926
	All	364.50 ± 292.64	259.74 ± 114.43	−104.76 ± 242.68	2.103_*t*_	0.057	415.25 ± 338.58	337.01 ± 173.33	−78.23 ± 309.60	−0.392_*t*_	0.695	0.114	0.737
Omega‐3 (g)	Female	0.74 ± 0.34	0.86 ± 0.78	0.12 ± 0.65	−0.533_*z*_	0.594	0.73 ± 0.24	1.05 ± 0.64	0.32 ± 0.56	−1.289_*t*_	0.254	0.824	0.372
	Male	1.56 ± 1.14	1.28 ± 0.59	−0.27 ± 0.64	1.700_*t*_	0.188	1.28 ± 0.56	1.51 ± 1.00	0.22 ± 1.06	−0.672_*t*_	0.531	1.584	0.224
	All	1.07 ± 0.85	1.03 ± 0.73	−0.04 ± 0.66	−0.105_*z*_	0.917	0.95 ± 0.48	1.23 ± 0.82	0.28 ± 0.78	−1.177_*z*_	0.239	2.416	0.127
Omega‐6 (g)	Female	8.97 ± 4.85	6.11 ± 5.35	−2.86 ± 5.75	1.520_*t*_	0.167	9.29 ± 5.61	12.87 ± 6.88	3.58 ± 8.77	−0.456_*t*_	0.668	**5.653**	**0.024** *
	Male	18.99 ± 12.74	10.68 ± 5.67	−8.32 ± 13.27	1.931_*t*_	0.149	17.10 ± 9.29	18.88 ± 9.77	1.78 ± 11.29	−1.363_*t*_	0.173	3.358	0.083
	All	12.98 ± 9.98	7.93 ± 5.83	−5.04 ± 9.63	2.399_*t*_	**0.034** *	12.42 ± 8.12	15.28 ± 8.51	2.86 ± 9.67	−1.467_*t*_	0.170	**8.382**	**0.006** **
Fiber (g)	Female	11.76 ± 5.03	20.41 ± 2.22	8.65 ± 5.80	−3.764_*t*_	**0.006** **	10.97 ± 5.01	10.74 ± 4.47	−0.23 ± 6.23	−0.014_*t*_	0.989	**21.024**	**< 0.001** **
	Male	19.21 ± 9.43	24.92 ± 6.65	5.70 ± 7.88	−5.265_*t*_	**0.013** *	12.77 ± 5.79	14.78 ± 6.32	2.01 ± 7.09	−0.581_*t*_	0.586	**9.129**	**0.007** **
	All	14.74 ± 7.87	22.22 ± 4.96	7.47 ± 6.71	−5.512_*t*_	**< 0.001** **	11.69 ± 5.29	12.36 ± 5.54	0.66 ± 6.54	−0.471_*t*_	0.647	**13.197**	**0.001** **

*Note:* Bold values indicate statistical significance at *p* < 0.05 (*) and *p* < 0.01 (**).

Abbreviations: *F*, repeated measures mixed‐way analysis of variance; SD, standard deviation; *t*, dependent samples *t*‐test value; *X̄*, mean; *z*, Wilcoxon signed‐rank test.

* *p* < 0.05.

** *p* < 0.01.

The hour‐based comparisons of the averages of hunger and satiety, which the participants evaluated subjectively at the beginning of the experiment and during the following 6 h, according to the hamburgers they ate, are given in Table 5. Participants felt statistically less hungry at Hours 4 and 5 (*p* = 0.013, *p* = 0.003, respectively) and more satiated at Hours 3, 4, and 5 (*p* = 0.025, *p* = 0.013, *p* = 0.029, respectively) when they ate PEHM compared to CHM. These statistically significant satiety results were also found when evaluating only men at Hour 3 (*p* = 0.024) and only women at Hour 4 (*p* = 0.047).

**TABLE 5:** Comparison of participants' subjective hunger and satiety evaluations according to the hamburgers consumed.

h	Sex	Subjective hunger						Subjective satiety					
		PEHM		CHM		*t*/*z*	*p*_1_	PEHM		CHM		*t*/*z*	*p*_2_
		*n*	*X̄* ± SD	*n*	*X̄* ± SD			*n*	*X̄* ± SD	*n*	*X̄* ± SD		
0	Female	15	70.83 ± 10.21	15	70.83 ± 10.21	0_*t*_	1	15	20.83 ± 7.72	15	20.00 ± 9.21	−0.107_*z*_	0.915
	Male	10	83.75 ± 14.49	10	83.75 ± 15.65	0_*t*_	1	10	10.00 ± 9.86	10	16.25 ± 10.29	−1.387_*t*_	0.182
	Together	25	76.00 ± 13.46	25	76.00 ± 13.94	0_*t*_	1	25	16.50 ± 10.03	25	18.50 ± 9.63	−0.719_*t*_	0.475
1	Female	15	12.50 ± 14.17	15	7.50 ± 13.19	1_*t*_	0.326	15	80.83 ± 18.82	15	86.67 ± 13.75	−0.969_*t*_	0.341
	Male	10	11.25 ± 7.10	10	20.00 ± 16.87	−1.512_*t*_	0.156	10	81.25 ± 14.73	10	76.25 ± 14.97	0.753_*t*_	0.461
	Together	25	12.00 ± 11.68	25	12.50 ± 15.73	−0.128_*t*_	0.899	25	81.00 ± 16.97	25	82.50 ± 14.88	−0.332_*t*_	0.741
2	Female	15	15.00 ± 15.81	15	12.50 ± 14.17	0.456_*t*_	0.652	15	82.50 ± 19.36	15	76.67 ± 18.22	0.850_*t*_	0.403
	Male	10	15.00 ± 12.91	10	28.75 ± 18.68	−1.915_*t*_	0.072	10	82.50 ± 15.81	10	67.50 ± 16.87	2.051_*t*_	0.055
	Together	25	15.00 ± 14.43	25	19.00 ± 17.72	−0.875_*t*_	0.386	25	82.50 ± 17.68	25	73.00 ± 17.93	1.887_*t*_	0.065
3	Female	15	21.67 ± 20.30	15	21.67 ± 15.28	0_*t*_	1	15	75.00 ± 23.15	15	68.33 ± 19.97	0.845_*t*_	0.405
	Male	10	17.50 ± 24.44	10	38.75 ± 25.99	−1.884_*t*_	0.076	10	82.50 ± 19.72	10	53.75 ± 31.21	2.463_*t*_	**0.024** *
	Together	25	20.00 ± 21.65	25	28.50 ± 21.51	−1.393_*t*_	0.170	25	78.00 ± 21.73	25	62.50 ± 25.52	2.312_*t*_	**0.025** *
4	Female	15	27.50 ± 20.70	14	43.75 ± 29.32	−1.734_*t*_	0.094	15	71.67 ± 24.31	14	49.11 ± 32.69	2.097_*t*_	**0.047** *
	Male	10	30.00 ± 23.72	10	52.50 ± 29.34	−1.886_*t*_	0.076	10	66.25 ± 25.03	10	48.75 ± 30.31	1.408_*t*_	0.176
	Together	25	28.50 ± 21.51	24	47.40 ± 29.02	**−2.518**_*t*_	**0.013** *	25	69.50 ± 24.23	24	48.96 ± 31.04	2.575_*t*_	**0.013** *
5	Female	15	37.50 ± 24.55	10	56.25 ± 18.87	−2.042_*t*_	0.053	15	56.67 ± 25.38	10	42.50 ± 27.76	1.317_*t*_	0.201
	Male	7	39.29 ± 16.81	8	67.19 ± 30.57	−2.141_*t*_	0.052	7	60.71 ± 15.19	8	34.38 ± 35.20	1.922_*t*_	0.084
	Together	22	38.07 ± 21.98	18	61.11 ± 24.59	**−3.127**_*t*_	**0.003** **	22	57.95 ± 22.34	18	38.89 ± 30.59	2.277_*t*_	**0.029** *
6	Female	15	58.33 ± 27.41	6	56.25 ± 17.23	0.172_*t*_	0.866	15	41.67 ± 26.59	6	39.58 ± 18.40	0.175_*t*_	0.863
	Male	5	55.00 ± 25.92	6	70.83 ± 31.29	−0.901_*t*_	0.391	5	42.50 ± 20.92	6	29.17 ± 31.29	0.810_*t*_	0.439
	Together	20	57.50 ± 26.41	12	63.54 ± 25.26	−0.637_*t*_	0.529	20	41.88 ± 24.76	12	34.38 ± 25.07	0.826_*t*_	0.415

*Note:* Bold values indicate statistical significance at *p* < 0.05 (*) and *p* < 0.01 (**).

Abbreviations: SD, standard deviation; *t*, dependent samples *t*‐test value; *X̄*, mean; *z*, Wilcoxon signed‐rank test.

* *p* < 0.05.

** *p* < 0.01.

## 4 Discussion

In the first part of the study, 5% and 7.5% PEHM were compared to CHM in terms of sensory factors, general appreciation, and hedonic scale scores. In a study where low‐fat chicken bologna sausages were enriched with inulin, oat fiber, and psyllium at 3% and 6% to develop them, it was observed that the scores of the sausages both in terms of factors and general appreciation decreased when psyllium was added (Ferjančič et al. 2021). In a study where chicken nuggets were enriched with psyllium at 2%, 4%, and 6% in addition to 10% rice bran, it was observed that the sensory analysis scores decreased as the fiber ratio increased, although the nuggets enriched with 10% rice bran and 4% psyllium were found to be organoleptically suitable (Mehta et al. 2013). A study that compared the sensory characteristics of pork sausages enriched with 0.5%–1%, 1%, and 2%–3% psyllium to those without enrichment showed that the desired characteristics increased in the sausages enriched with 0.5%–1% and 1% psyllium compared to control sausages, but this trend reversed when the enrichment level reached 2%–3% (Zhou et al. 2019). In a study where psyllium was added to pork and beef mixture sausage at 2%, 4%, 6%, and 8%, it was shown that the organoleptic properties of the final product could be enriched by 2% without any change (Danyliv et al. 2019). In this study, although the CHM scored higher in terms of taste in the first block of the sensory analysis panel, no difference was observed in the second block. When comparing the blocks, the hamburgers were found to be similar in terms of sensory factors, general preference, and hedonic appreciation, and it was deemed appropriate to use hamburgers enriched with 7.5% psyllium during the experimental period.

In the second part of the study, the changes in postprandial lipidemia and glycemia, prospective food intake, and subjective hunger and satiety ratings of participants who consumed PEHM and CHM were compared.

Our primary finding regarding lipidemia was that the consumption of PEHM significantly attenuated the postprandial increase in triglycerides and VLDL compared to CHM. This result is consistent with psyllium's established mechanisms; the viscous gel it forms in the gut can delay the activity of digestive enzymes and trap dietary lipids, thereby slowing their absorption and reducing the subsequent rise in postprandial blood lipids (Narayanan and Pitchumoni 2020). However, no statistically significant differences were found between the two hamburgers in terms of total cholesterol, LDL, or HDL levels. This aligns with a similar acute crossover intervention study by Khossousi et al. (2008), where a 12 g of psyllium consumption just before the meal significantly lowered postprandial triglyceride levels compared to control with no significant differences in total cholesterol, LDL, or HDL levels. This could be attributed to the short‐term nature of the intervention, as psyllium's effects on cholesterol metabolism typically become more pronounced with sustained intake. Moreover, the high fat content of the hamburgers might have overshadowed psyllium's effects, with the body prioritizing fat digestion. A single 12 g dose of psyllium may not be sufficient to induce significant lipid profile changes, and individual variations in metabolism may have influenced the outcomes. Indeed, the most significant benefits of psyllium on total cholesterol and LDL are typically observed in longer term studies where mechanisms like increased bile acid excretion (Narayanan and Pitchumoni 2020) become more impactful, as demonstrated in trials lasting from three to twelve months. McRorie Jr. et al. (2017) found that daily supplementation with 3.4 g psyllium before meals led to significant reductions in LDL and total cholesterol over 3 months compared to wheat dextrin. Similarly, Pal et al. (2017) reported significantly reduced total cholesterol, LDL, and triglyceride levels after 12 months of psyllium intake in overweight and obese individuals. In a 3‐month trial, Asghar et al. (2011) also observed decreases in total cholesterol, LDL, and triglycerides, along with an increase in HDL, among hyperlipidemic individuals receiving 10 g/day psyllium.

An intriguing finding in our study was the postprandial reduction in HDL and LDL cholesterol concentrations compared to fasting levels, even following consumption of the control hamburger. This phenomenon is consistent with established physiological responses to high‐fat meals. As shown by Wojczynski et al. (2011), dietary fat intake triggers an upregulation of cholesteryl ester transfer protein (CETP), which facilitates the exchange of cholesterol esters from HDL and LDL to newly formed chylomicrons. This process, coupled with increased hepatic lipase activity that further catabolizes these lipoproteins, leads to an accelerated clearance of HDL and LDL from the plasma, resulting in temporarily lower concentrations. This well‐documented mechanism (Rizi et al. 2019; Shah et al. 2018, 2017) supports the changes observed even after CHM consumption and illustrates the dynamic nature of postprandial lipoprotein metabolism.

Similarly, the decrease in blood glucose at the 2‐h mark can be understood as a normal physiological excursion. Fasting glucose levels in the morning are often slightly elevated relative to other times of the day due to the “dawn phenomenon,” where early‐morning hormonal surges, particularly in cortisol and growth hormone, increase hepatic glucose output to prepare the body for daily activity (Schmidt et al. 1981). Following a morning meal, healthy individuals typically exhibit high insulin sensitivity, leading to a rapid and efficient glucose disposal (Saad et al. 2012). The combination of this robust insulin response acting on a slightly elevated baseline can result in a temporary glucose decrease below the initial fasting value before homeostatic mechanisms restore balance (Jarvis et al. 2023). This pattern is consistent with findings from other trials, such as Fletcher et al. (2018), who observed a similar glucose nadir below baseline approximately 2 h after a standard‐energy meal in resting individuals.

Regarding postprandial glycemia, the consumption of PEHM resulted in a statistically significant reduction in 2‐h blood glucose levels compared to the control. This finding is supported by literature demonstrating psyllium's efficacy when integrated into a food matrix. For instance, Brennan et al. (2012) found that a psyllium‐enriched snack effectively reduced glucose peaks, rather than delaying them as an oat‐enriched snack did, highlighting the distinct physiological action of psyllium. Furthermore, McRorie Jr. et al. (2017) attributed psyllium's superior glycemic control over wheat dextrin to its non‐fermentable nature, which prevents energy harvest from the colon. The design of our study, where psyllium was an intrinsic component of the meal, likely enhanced its effectiveness. This aligns with the hypothesis from Khossousi et al. (2008), who found no significant effect with a premeal psyllium supplement and suggested that direct incorporation into food would be a more potent strategy for glycemic control.

In contrast to the effect on glucose, the observed trend toward lower postprandial insulin concentrations and HOMA‐IR values with PEHM did not reach statistical significance. This should be interpreted within the context of our study's acute design, which focused on the immediate postprandial response. In fact, our results mirror those of another acute intervention by Khossousi et al. (2008), who also reported that while the postprandial increase in insulin was lower following psyllium consumption compared to a control meal, the difference was not statistically significant. This finding from short‐term studies differs from the results of long‐term trials, such as Pal et al. (2017), who reported significantly lower fasting insulin and HOMA‐IR levels in participants after long‐term daily consumption of psyllium for 3, 6, and 12 months. This discrepancy highlights the critical difference between acute and chronic effects: while consistent, long‐term intake appears to improve baseline insulin sensitivity, a single dose may not be sufficient to significantly blunt the acute insulin secretory response to a meal, particularly in a healthy, euglycemic population. This is consistent with the observation by Pal et al. (2017) that psyllium's effects are often proportional to an individual's baseline glycemic status. Moreover, it is crucial to acknowledge that the participants' fasting insulin levels were significantly higher on the first trial day compared to the second. Although the crossover design is intended to mitigate such order effects, this baseline difference warrants a cautious interpretation of the nonsignificant insulin‐related findings.

A key outcome of this trial was that the PEHM not only influenced subjective appetite but also led to a significant reduction in subsequent ad libitum energy, total fat, PUFA, and omega‐6 intake for the remainder of the day. This acute finding provides a potential mechanism for the benefits observed in longer‐term studies. For example, trials have shown that daily psyllium consumption over several months results in lower overall energy and fat intake, aiding in weight management (Pal et al. 2017; Pal et al. 2011). Our results suggest that this chronic effect is likely the cumulative result of immediate, day‐by‐day influences on satiety and hunger that guide subsequent food choices.

This behavioral change in food intake was directly supported by the participants' subjective ratings. While both the PEHM and CHM induced initial fullness, the PEHM was significantly more effective at prolonging satiety and suppressing the return of hunger in the hours following consumption. This sustained effect is consistent with psyllium's ability to form a viscous gel that delays gastric emptying (Fischer et al. 2004). It is noteworthy that the literature on psyllium's acute effects on appetite has shown some variability. While studies by Khossousi et al. (2008) and Karhunen et al. (2010) observed trends toward reduced hunger and increased satiety, these differences did not reach statistical significance. In contrast, our significant findings are consistent with a dose–response trial by Brum et al. (2016), which found significant effects at doses of 6.8 g and higher. The 12 g dose used in our study falls within this effective range, and its incorporation into a complex, high‐energy food matrix may also have contributed to the pronounced effect observed. The consistency between the subjective reports of prolonged satiety and the objective measure of reduced energy consumption indicates that enriching commonly consumed foods with psyllium can be a practical strategy to aid in appetite and energy regulation.

The results of this study suggest that 7.5% (12 g) psyllium per serving is acceptable to consumers. To scale psyllium enrichment for commercial fast food, different dosages could be tested through large consumer panels to evaluate acceptability and sensory characteristics. Integrating psyllium into widely consumed fast food products could contribute to improving public health, particularly among younger populations. By offering a simple and effective way to increase fiber intake, this approach could support efforts to reduce the risks associated with chronic diseases such as cardiovascular disease and obesity, promoting overall health and well‐being.

## 5 Limitations

This study has several limitations. First, the sample consisted of young, healthy adults (aged 19–35 years, BMI 18.5–25 kg/m^2^), which restricts the generalizability of the findings to populations who might benefit more from fiber enrichment, such as individuals with obesity, diabetes, or elderly adults. Second, the study evaluated only the acute effects of psyllium‐enriched hamburger consumption; therefore, the long‐term metabolic impacts and the sustainability of incorporating psyllium into the diet remain unknown. Finally, participants were recruited via social media platforms, which may introduce selection bias and limit the representativeness of the sample compared to the general population. Furthermore, while our final sample size (*n* = 25) exceeded the a priori requirement of 18 participants and was confirmed by a post hoc analysis to have sufficient power, the attrition of three participants post‐randomization (two for dyslipidemia, one for a lab error) is a noted limitation. The gender‐stratified analyses presented were exploratory and should be interpreted with caution; the study's primary conclusions are based on the main effects observed in the entire sample.

## 6 Conclusions

In this study, we enriched HM with psyllium to significantly enhance their fiber content and found that their sensory properties remained highly acceptable even at a 7.5% enrichment level. The consumption of psyllium‐enriched hamburgers resulted in a notably more moderate postprandial increase in triglyceride and VLDL levels compared to control hamburgers, suggesting a potential reduction in cardiovascular risk. Moreover, lower postprandial blood glucose levels, decreased prospective food intake, and improved subjective appetite ratings—characterized by reduced hunger and increased satiety—were consistently observed. The consumption of 12 g of psyllium with a meal was well tolerated by participants, with no adverse effects reported.

These findings highlight the promising potential of psyllium enrichment to positively influence both short‐term metabolic responses and appetite regulation in healthy adults. Given the widespread issue of low dietary fiber intake in populations with high fast‐food consumption, fortifying commonly consumed foods like hamburgers with psyllium could be an effective, practical strategy to help meet daily fiber intake recommendations. However, additional long‐term studies across diverse populations are essential to establish the sustainability of these benefits and to explore their broader metabolic implications.

In the context of commercial application, it is essential to ensure that psyllium‐enriched products comply with existing food safety and nutritional regulations. Given the patent registration, the formulation and production processes would need to meet national and international food standards regarding fiber fortification, allergen labeling, and health claims. Regulatory approval processes would be necessary to confirm the safety, efficacy, and labeling requirements before large‐scale integration into the fast food industry. Additionally, considerations regarding the cost‐effectiveness of psyllium‐enriched products, as well as consumer acceptance, would play crucial roles in the commercial success and scalability of such innovations in the fast food sector.

## 7 Suggestions for Future Studies

Upon review of the literature, psyllium is often studied in individuals who are overweight, obese, or within the normal range of BMI. Therefore, there is a need for further research to examine the appropriateness of using psyllium, particularly in thin individuals due to its potential anorectic effects. Additionally, individuals at risk of micronutrient deficiencies should be closely monitored, and their nutrition programs should be planned accordingly in long‐term and high‐dose psyllium use, as reduced food intake is associated with decreased intake of vitamins and minerals.

Future research should also investigate the effects of psyllium enrichment on different food matrices beyond hamburgers to evaluate its applicability across various dietary patterns. Moreover, studies involving clinical populations such as individuals with obesity, diabetes, or cardiovascular risk factors are warranted to better understand the therapeutic potential of psyllium. Mechanistic studies exploring its impact on gut microbiota composition and function would further clarify the pathways through which psyllium exerts its metabolic benefits.

## Author Contributions

**Ahmet Murat Günal:** conceptualization (equal), data curation (lead), formal analysis (lead), investigation (lead), methodology (equal), writing – original draft (lead), writing – review and editing (equal). **Hande Öngün Yılmaz:** conceptualization (equal), methodology (equal), supervision (lead), writing – review and editing (lead). **Murat Baş:** conceptualization (equal), methodology (equal), supervision (lead), writing – review and editing (supporting).

## Ethics Statement

The study adhered to the guidelines outlined in the Declaration of Helsinki and received approval from the Acıbadem Mehmet Ali Aydınlar University Medical Research Evaluation Board's (ATADEK) ethics committee (2021‐24/31).

## Consent

Participation in the study is based on voluntariness. Written informed consent was obtained from all study participants.

## Conflicts of Interest

The authors declare no conflicts of interest.

## Supporting information


**Tables S1–S2:** fsn371066‐sup‐0001‐TableS1‐S2.docx.

## Data Availability

The data that support the findings of this study are available on request from the corresponding author. The data are not publicly available due to privacy or ethical restrictions.

## References

[fsn371066-bib-0001] Åberg, S. , M. Palmnäs‐Bédard , T. Karlsson , T. Hjorth , K. N. Iversen , and R. Landberg . 2023. “Evaluation of Subjective Appetite Assessment Under Free‐Living vs. Controlled Conditions: A Randomized Crossover Trial Comparing Whole‐Grain Rye and Refined Wheat Diets (VASA‐Home).” Nutrients 15, no. 11: 2456. 10.3390/NU15112456/S1. 37299419 PMC10254760

[fsn371066-bib-0002] Asghar, J. , A. Bashir , M. Aslam , S. Asif , A. Chaudhry , and S. Murad . 2011. “Single Blind and Placebo Controlled Research Study of Effects of Ispaghula on Serum Lipids.” Pakistan Journal of Medical & Health Sciences 5: 654–657.

[fsn371066-bib-0048] BeBiS . n.d. “Beslenme Bilgi Sistemi (Version 8.0).” In Ebispro for Windows, Stuttgart, Germany (8.0).

[fsn371066-bib-0003] Brennan, M. A. , E. J. Derbyshire , C. S. Brennan , and B. K. Tiwari . 2012. “Impact of Dietary Fibre‐Enriched Ready‐To‐Eat Extruded Snacks on the Postprandial Glycaemic Response of Non‐Diabetic Patients.” Molecular Nutrition & Food Research 56, no. 5: 834–837. 22648629 10.1002/mnfr.201100760

[fsn371066-bib-0004] Brum, J. M. , R. D. Gibb , J. C. Peters , and R. D. Mattes . 2016. “Satiety Effects of Psyllium in Healthy Volunteers.” Appetite 105: 27–36. 27166077 10.1016/j.appet.2016.04.041

[fsn371066-bib-0005] Danyliv, M. M. , O. A. Vasilenko , O. N. Ozherelyeva , and Y. A. Shestakova . 2019. “Improvement of Sausage Production Technology.” IOP Conference Series: Earth and Environmental Science 341, no. 1: e012131.

[fsn371066-bib-0006] Drapeau, V. , J. Blundell , F. Therrien , C. Lawton , D. Richard , and A. Tremblay . 2005. “Appetite Sensations as a Marker of Overall Intake.” British Journal of Nutrition 93, no. 2: 273–280. 15788121 10.1079/bjn20041312

[fsn371066-bib-0007] Dwan, K. , T. Li , D. G. Altman , and D. Elbourne . 2019. “CONSORT 2010 Statement: Extension to Randomised Crossover Trials.” British Medical Journal 366: l4378. 10.1136/BMJ.L4378. 31366597 PMC6667942

[fsn371066-bib-0008] FDA . 1998. “FDA Allows Foods Containing Psyllium to Make Health Claim on Reducing Risk of Heart Disease.” Rockville, MD: US Department of Health and Human Services.

[fsn371066-bib-0009] Ferjančič, B. , S. Kugler , M. Korošec , T. Polak , and J. Bertoncelj . 2021. “Development of Low‐Fat Chicken Bologna Sausages Enriched With Inulin, Oat Fibre or Psyllium.” International Journal of Food Science & Technology 56, no. 4: 1818–1828.

[fsn371066-bib-0010] Fischer, M. H. , N. Yu , G. R. Gray , J. Ralph , L. Anderson , and J. A. Marlett . 2004. “The Gel‐Forming Polysaccharide of Psyllium Husk ( *Plantago ovata* Forsk).” Carbohydrate Research 339, no. 11: 2009–2017. 15261594 10.1016/j.carres.2004.05.023

[fsn371066-bib-0011] Fletcher, E. A. , J. Salmon , S. A. McNaughton , et al. 2018. “Effects of Breaking Up Sitting on Adolescents' Postprandial Glucose After Consuming Meals Varying in Energy: A Cross‐Over Randomised Trial.” Journal of Science and Medicine in Sport 21, no. 3: 280–285. 10.1016/J.JSAMS.2017.06.002. 28625540

[fsn371066-bib-0012] Gibb, R. D. , J. W. McRorie Jr. , D. A. Russell , V. Hasselblad , and D. A. D'Alessio . 2015. “Psyllium Fiber Improves Glycemic Control Proportional to Loss of Glycemic Control: A Meta‐Analysis of Data in Euglycemic Subjects, Patients at Risk of Type 2 Diabetes Mellitus, and Patients Being Treated for Type 2 Diabetes Mellitus.” American Journal of Clinical Nutrition 102, no. 6: 1604–1614. 26561625 10.3945/ajcn.115.106989

[fsn371066-bib-0013] Gibbons, C. , M. Hopkins , K. Beaulieu , P. Oustric , and J. E. Blundell . 2019. “Issues in Measuring and Interpreting Human Appetite (Satiety/Satiation) and Its Contribution to Obesity.” Current Obesity Reports 8, no. 2: 77–87. 31037612 10.1007/s13679-019-00340-6 PMC6517339

[fsn371066-bib-0014] Howarth, N. C. , E. Saltzman , and S. B. Roberts . 2001. “Dietary Fiber and Weight Regulation.” Nutrition Reviews 59, no. 5: 129–139. 11396693 10.1111/j.1753-4887.2001.tb07001.x

[fsn371066-bib-0015] InMobi . 2023. “Most Ordered Fast Food US 2023|Statista.” Statista Website. https://www.statista.com/statistics/1414293/most‐ordered‐fast‐food‐us/.

[fsn371066-bib-0016] Isganaitis, E. , and R. H. Lustig . 2005. “Fast Food, Central Nervous System Insulin Resistance, and Obesity.” Arteriosclerosis, Thrombosis, and Vascular Biology 25, no. 12: 2451–2462. 16166564 10.1161/01.ATV.0000186208.06964.91

[fsn371066-bib-0017] Jarvis, P. R. E. , J. L. Cardin , P. M. Nisevich‐Bede , and J. P. McCarter . 2023. “Continuous Glucose Monitoring in a Healthy Population: Understanding the Post‐Prandial Glycemic Response in Individuals Without Diabetes Mellitus.” Metabolism 146: 155640. 10.1016/J.METABOL.2023.155640. 37356796

[fsn371066-bib-0018] Karhunen, L. J. , K. R. Juvonen , S. M. Flander , et al. 2010. “A Psyllium Fiber‐Enriched Meal Strongly Attenuates Postprandial Gastrointestinal Peptide Release in Healthy Young Adults.” Journal of Nutrition 140, no. 4: 737–744. 20147463 10.3945/jn.109.115436

[fsn371066-bib-0019] Khossousi, A. , C. W. Binns , S. S. Dhaliwal , and S. Pal . 2008. “The Acute Effects of Psyllium on Postprandial Lipaemia and Thermogenesis in Overweight and Obese Men.” British Journal of Nutrition 99, no. 5: 1068–1075. 18005484 10.1017/S0007114507864804

[fsn371066-bib-0020] Lo, Y. M. 2022. “The ART and LOGIC of Scholarly Communication: Effective Skills for Publication and Beyond.” Food Science & Nutrition 10, no. 4: 981–984. 10.1002/FSN3.2766. 35432967 PMC9007295

[fsn371066-bib-0021] Matthews, D. R. , J. P. Hosker , A. S. Rudenski , B. A. Naylor , D. F. Treacher , and R. C. Turner . 1985. “Homeostasis Model Assessment: Insulin Resistance and β‐Cell Function From Fasting Plasma Glucose and Insulin Concentrations in Man.” Diabetologia 28, no. 7: 412–419. 10.1007/BF00280883. 3899825

[fsn371066-bib-0022] McRorie, J. W., Jr. 2015. “Evidence‐Based Approach to Fiber Supplements and Clinically Meaningful Health Benefits, Part 1: What to Look for and How to Recommend an Effective Fiber Therapy.” Nutrition Today 50, no. 2: 82. 25972618 10.1097/NT.0000000000000082 PMC4415962

[fsn371066-bib-0023] McRorie, J. W., Jr. , R. D. Gibb , J. B. Womack , and D. J. Pambianco . 2017. “Psyllium Is Superior to Wheat Dextrin for Lowering Elevated Serum Cholesterol.” Nutrition Today 52, no. 6: 289–294.

[fsn371066-bib-0024] Mehta, N. , S. S. Ahlawat , D. P. Sharma , S. Yadav , and D. Arora . 2013. “Sensory Attributes of Chicken Meat Rolls and Patties Incorporated With the Combination Levels of Rice Bran and Psyllium Husk.” Journal of Animal Research 3, no. 2: 179–185.

[fsn371066-bib-0025] Narayanan, S. , and C. S. Pitchumoni . 2020. “Dietary Fiber.” In Geriatric Gastroenterology, 1–16. Springer International Publishing.

[fsn371066-bib-0049] National Institutes of Health (NIH) . n.d.‐a. “24‐hour Dietary Recall (24HR) At a Glance|Dietary Assessment Primer.” Https://Dietassessmentprimer.Cancer.Gov/Profiles/Recall. Retrieved January 13, 2025, from https://dietassessmentprimer.cancer.gov/profiles/recall/index.html.

[fsn371066-bib-0050] National Institutes of Health (NIH) . n.d.‐b. “Food Record at a Glance|Dietary Assessment Primer.” Https://Dietassessmentprimer.Cancer.Gov/Profiles/Record. Retrieved January 13, 2025, from https://dietassessmentprimer.cancer.gov/profiles/record/index.html.

[fsn371066-bib-0026] OpinionWay . 2019. “Share of French People Who Like Fast Food Restaurants by Age 2019|Statista.” Statista Website. https://www.statista.com/statistics/1026980/people‐who‐like‐fast‐foods‐by‐age‐france/.

[fsn371066-bib-0027] Pal, S. , S. Ho , R. J. Gahler , and S. Wood . 2017. “Effect on Insulin, Glucose and Lipids in Overweight/Obese Australian Adults of 12 Months Consumption of Two Different Fibre Supplements in a Randomised Trial.” Nutrients 9, no. 2: 91. 28146065 10.3390/nu9020091 PMC5331522

[fsn371066-bib-0028] Pal, S. , A. Khossousi , C. Binns , S. Dhaliwal , and V. Ellis . 2011. “The Effect of a Fibre Supplement Compared to a Healthy Diet on Body Composition, Lipids, Glucose, Insulin and Other Metabolic Syndrome Risk Factors in Overweight and Obese Individuals.” British Journal of Nutrition 105, no. 1: 90–100. 20727237 10.1017/S0007114510003132

[fsn371066-bib-0029] Parker, B. A. , K. Sturm , C. G. MacIntosh , C. Feinle , M. Horowitz , and I. M. Chapman . 2004. “Relation Between Food Intake and Visual Analogue Scale Ratings of Appetite and Other Sensation in Healthy Older and Young Subjects.” European Journal of Clinical Nutrition 58, no. 2: 212–218. 10.1038/SJ.EJCN.1601768. 14749739

[fsn371066-bib-0030] Phan, J. L. , J. M. Cowley , K. A. Neumann , L. Herliana , L. A. O'Donovan , and R. A. Burton . 2020. “The Novel Features of *Plantago ovata* Seed Mucilage Accumulation, Storage and Release.” Scientific Reports 10, no. 1: 11766. 32678191 10.1038/s41598-020-68685-w PMC7366641

[fsn371066-bib-0031] Rizi, E. P. , S. Baig , T. P. Loh , S. A. Toh , C. M. Khoo , and E. Shyong Tai . 2019. “Two‐Hour Postprandial Lipoprotein Particle Concentration Differs Between Lean and Obese Individuals.” Frontiers in Physiology 10: 856. 10.3389/FPHYS.2019.00856/FULL. 31379592 PMC6649689

[fsn371066-bib-0046] Saad, A. , C. D. Man , D. K. Nandy , et al. 2012. “Diurnal Pattern to Insulin Secretion and Insulin Action in Healthy Individuals.” Diabetes 61, no. 11: 2691–2700. 10.2337/DB11-1478. 22751690 PMC3478548

[fsn371066-bib-0032] Schmidt, M. I. , A. Hadji‐Georgopoulos , M. Rendell , S. Margolis , and A. Kowarski . 1981. “The Dawn Phenomenon, an Early Morning Glucose Rise: Implications for Diabetic Intraday Blood Glucose Variation.” Diabetes Care 4, no. 6: 579–585. 10.2337/DIACARE.4.6.579. 6751733

[fsn371066-bib-0033] Schulz, K. F. , D. G. Altman , and D. Moher . 2010. “CONSORT 2010 Statement: Updated Guidelines for Reporting Parallel Group Randomised Trials.” Journal of Pharmacology and Pharmacotherapeutics 1, no. 2: 100–107. 21350618 10.4103/0976-500X.72352 PMC3043330

[fsn371066-bib-0034] Shah, M. , B. Adams‐Huet , B. Franklin , M. Phillips , and J. Mitchell . 2018. “The Effects of High‐Protein and High‐Monounsaturated Fat Meals on Postprandial Lipids, Lipoprotein Particle Numbers, Cytokines, and Leptin Responses in Overweight/Obese Subjects.” Metabolic Syndrome and Related Disorders 16, no. 3: 150–158. 10.1089/MET.2017.0167. 29596044

[fsn371066-bib-0035] Shah, M. , M. Jaffery , B. Adams‐Huet , B. Franklin , J. Oliver , and J. Mitchell . 2017. “Effect of Meal Composition on Postprandial Lipid Concentrations and Lipoprotein Particle Numbers: A Randomized Cross‐Over Study.” PLoS One 12, no. 2: e0172732. 10.1371/JOURNAL.PONE.0172732. 28222178 PMC5319704

[fsn371066-bib-0036] Singh, A. , D. Dhanasekaran , N. Ganamurali , L. Preethi , and S. Sabarathinam . 2021. “Junk Food‐Induced Obesity—A Growing Threat to Youngsters During the Pandemic.” Obesity Medicine 26: 100364. 34580647 10.1016/j.obmed.2021.100364 PMC8459649

[fsn371066-bib-0045] Suter, P. M. , G. Marmier , C. Veya‐Linder , et al. 2005. “Effect of Orlistat on Postprandial Lipemia, NMR Lipoprotein Subclass Profiles and Particle Size.” Atherosclerosis 180, no. 1: 127–135. 15823285 10.1016/j.atherosclerosis.2004.11.023

[fsn371066-bib-0038] TR Ministry of Health . 2019. “Türkiye Beslenme ve Sağlik Araştirmasi (TBSA) 2017.” https://hsgm.saglik.gov.tr/depo/birimler/saglikli‐beslenme‐ve‐hareketli‐hayat‐db/Dokumanlar/Kitaplar/Turkiye_Beslenme_ve_Saglik_Arastirmasi_TBSA_2017.pdf.

[fsn371066-bib-0037] TR Ministry of Health . 2022. “Türkiye Beslenme Rehberi.” Ankara. https://hsgm.saglik.gov.tr/depo/birimler/saglikli‐beslenme‐ve‐hareketli‐hayat‐db/Dokumanlar/Rehberler/Turkiye_Beslenme_Rehber_TUBER_2022_min.pdf.

[fsn371066-bib-0039] Trumbo, P. , S. Schlicker , A. A. Yates , and M. Poos . 2002. “Dietary Reference Intakes for Energy, Carbohydrate, Fiber, Fat, Fatty Acids, Cholesterol, Protein and Amino Acids.” Journal of the American Dietetic Association 102, no. 11: 1621–1631. 12449285 10.1016/s0002-8223(02)90346-9

[fsn371066-bib-0040] U.Di.Con . 2024. “Frequency of Fast Food Consumption by Age Italy 2024|Statista.” Statista Website. https://www.statista.com/statistics/1479924/fast‐food‐consumption‐frequency‐by‐age‐in‐italy/.

[fsn371066-bib-0041] Wanders, A. J. , J. J. G. C. van den Borne , C. de Graaf , et al. 2011. “Effects of Dietary Fibre on Subjective Appetite, Energy Intake and Body Weight: A Systematic Review of Randomized Controlled Trials.” Obesity Reviews 12, no. 9: 724–739. 21676152 10.1111/j.1467-789X.2011.00895.x

[fsn371066-bib-0042] Wojczynski, M. K. , S. P. Glasser , A. Oberman , et al. 2011. “High‐Fat Meal Effect on LDL, HDL, and VLDL Particle Size and Number in the Genetics of Lipid‐Lowering Drugs and Diet Network (GOLDN): An Interventional Study.” Lipids in Health and Disease 10, no. 1: 1–11. 10.1186/1476-511X-10-181/FIGURES/6. PMC3206850 22008512

[fsn371066-bib-0043] YouGov . 2025. “Most Popular American Food Dishes US Q4 2024|Statista.” Statista Website. https://www.statista.com/statistics/1351146/most‐popular‐american‐dishes‐in‐the‐us/.

[fsn371066-bib-0044] Zhou, Y. , L. Ma , Y. Yu , H. Zhu , H. Wang , and Y. Zhang . 2019. “Effect of Psyllium Husk Powder Addition on Quality of Meat Patties.” Journal of Food Science and Technology 37, no. 5: 42–49.

