# Supplementary material for: Enrichment of Hamburger Meatballs With Psyllium: Effects on Postprandial Lipidemia, Glycemia, Appetite, and Food Intake in a Triple‐Blind Randomized Controlled Crossover Trial
Source: Food Sci Nutr. 2025 Oct 9;13(10):e71066. doi: 10.1002/fsn3.71066 (PMC12509172; doi:10.1002/fsn3.71066)
Supplement: Supplementary file 1 — Tables S1–S2: fsn371066‐sup‐0001‐TableS1‐S2.docx. [file FSN3-13-e71066-s001.docx]

**Supporting Information**

**SuppTable 1.** Comparison of Participants’ Fasting Biochemical Measurements on Trial Days

| **Measurement** | **Sex** | **First Period** | **Second Period** | **t** | **p** |
| --- | --- | --- | --- | --- | --- |
|  |  | **X̄±SD** | **X̄±SD** |  |  |
| **Total Cholesterol**  **(mg/dL)** | Female | 167.77±22.97 | 167.82±24.66 | -0.005 | 0.996 |
|  | Male | 175.83±24.37 | 167.53±27.44 | 0.715 | 0.484 |
|  | All | 171.00±23.39 | 167.70±25.24 | 0.478 | 0.635 |
| **HDL**  **(mg/dL)** | Female | 70.10±13.26 | 65.33±12.72 | 1.006 | 0.323 |
|  | Male | 55.73±10.47 | 52.47±7.54 | 0.799 | 0.435 |
|  | All | 64.35±13.97 | 60.18±12.53 | 1.110 | 0.272 |
| **LDL**  **(mg/dL)** | Female | 83.63±24.6 | 88.32±25.39 | -0.514 | 0.612 |
|  | Male | 98.38±18.9 | 97.56±25.84 | 0.081 | 0.936 |
|  | All | 89.53±23.26 | 92.02±25.45 | -0.360 | 0.720 |
| **VLDL**  **(mg/dL)** | Female | 14.04±3.51 | 14.17±4.63 | -0.089 | 0.930 |
|  | Male | 21.72±12.52 | 17.50±8.43 | 0.884 | 0.388 |
|  | All | 17.11±8.99 | 15.50±6.47 | 0.726 | 0.471 |
| **Triglyceride**  **(mg/dL)** | Female | 70.20±17.55 | 70.87±23.14 | -0.089 | 0.930 |
|  | Male | 108.20±61.95 | 87.50±42.13 | 0.874 | 0.394 |
|  | All | 85.40±44.50 | 77.52±32.36 | 0.716 | 0.477 |
| **Glucose**  **(mg/dL)** | Female | 95.00±4.61 | 93.47±5.54 | 0.824 | 0.417 |
|  | Male | 98.50±7.88 | 98.80±7.83 | -0.085 | 0.933 |
|  | All | 96.40±6.22 | 95.60±6.93 | 0.429 | 0.670 |
| **Insulin**  **(μIU/mL)** | Female | 6.15±1.66 | 5.01±1.63 | 1.897 | 0.068 |
|  | Male | 7.90±2.54 | 6.45±2.62 | 1.257 | 0.225 |
|  | All | 6.85±2.19 | 5.58±2.15 | **2.057** | **0.045*** |
| **HOMA-IR** | Female | 1.45±0.42 | 1.16±0.39 | 1.965 | 0.059 |
|  | Male | 1.93±0.65 | 1.61±0.78 | 1.001 | 0.330 |
|  | All | 1.64±0.57 | 1.34±0.60 | 1.826 | 0.074 |

X̄: Mean; SD: Standard Deviation; t: Independent samples t-test value; *: p<0.05

**SuppTable 2.** Comparison of Participants’ Energy and Macronutrient Intakes on Pre-Trial Days

|  | **Sex** | **First Period** | **Second Period** | **t / z** | **p** |
| --- | --- | --- | --- | --- | --- |
|  |  | **X̄±SD** | **X̄±SD** |  |  |
| **Energy**  **(kcal)** | Female | 1379.82±436.06 | 1313.73±247.81 | 0.510^t^ | 0.615 |
|  | Male | 1925.71±714.41 | 1915.34±552.12 | 0.036^t^ | 0.971 |
|  | All | 1598.18±613.85 | 1554.38±490.53 | 0.279^t^ | 0.782 |
| **Carbohydrate**  **(g)** | Female | 157.98±70.94 | 137.70±28.00 | 1.030^t^ | 0.312 |
|  | Male | 191.73±81.96 | 173.39±71.92 | 0.532^t^ | 0.602 |
|  | All | 171.48±75.76 | 151.98±52.11 | 1.060^t^ | 0.295 |
| **Protein**  **(g)** | Female | 48.89±16.85 | 54.38±15.86 | -0.919^t^ | 0.366 |
|  | Male | 92.50±37.51 | 98.38±30.86 | -0.383^t^ | 0.707 |
|  | All | 66.33±34.19 | 71.98±31.43 | -0.608^t^ | 0.546 |
| **Total Fat**  **(g)** | Female | 59.64±21.18 | 59.05±19.00 | 0.080^t^ | 0.937 |
|  | Male | 84.77±38.72 | 90.02±23.71 | -0.366^z^ | 0.72 |
|  | All | 69.69±31.33 | 71.44±25.71 | -0.650^z^ | 0.516 |
| **SFA**  **(g)** | Female | 24.95±9.82 | 23.74±8.92 | 0.353^t^ | 0.727 |
|  | Male | 29.69±13.42 | 31.23±10.99 | -0.281^t^ | 0.782 |
|  | All | 26.84±11.37 | 26.74±10.28 | 0.035^t^ | 0.972 |
| **MUFA**  **(g)** | Female | 21.79±10.15 | 20.71±7.72 | 0.327^t^ | 0.747 |
|  | Male | 28.56±11.93 | 34.49±10.65 | -1.172^t^ | 0.257 |
|  | All | 24.50±11.18 | 26.22±11.17 | -0.546^t^ | 0.588 |
| **PUFA**  **(g)** | Female | 9.09±3.79 | 11.22±6.68 | -1.074^t^ | 0.292 |
|  | Male | 20.83±14.64 | 18.92±8.53 | 0.357^z^ | 0.726 |
|  | All | 13.79±11.11 | 14.30±8.26 | -0.631^z^ | 0.528 |
| **Cholesterol**  **(mg)** | Female | 207.65±132.12 | 277.13±203.30 | -1.110^t^ | 0.278 |
|  | Male | 662.89±445.26 | 559.30±238.10 | 0.649^t^ | 0.527 |
|  | All | 389.74±369.24 | 390.00±255.49 | -0.592^z^ | 0.554 |
| **Omega-3**  **(g)** | Female | 0.74±0.24 | 0.73±0.35 | 0.035^t^ | 0.973 |
|  | Male | 1.43±1.19 | 1.41±0.50 | 0.071^z^ | 0.944 |
|  | All | 1.02±0.83 | 1.00±0.53 | -0.475^z^ | 0.635 |
| **Omega-6**  **(g)** | Female | 8.11±3.70 | 10.15±6.25 | -1.092^t^ | 0.284 |
|  | Male | 18.84±13.38 | 17.25±8.37 | 0.318^z^ | 0.755 |
|  | All | 12.40±10.20 | 12.99±7.85 | -0.689^z^ | 0.491 |
| **Fiber**  **(g)** | Female | 12.12±4.33 | 10.61±5.54 | 0.831^t^ | 0.413 |
|  | Male | 18.13±10.41 | 13.85±5.19 | 1.164^t^ | 0.259 |
|  | All | 14.53±7.79 | 11.91±5.53 | 1.371^t^ | 0.177 |

X̄: Mean; SD: Standard Deviation; t: Independent samples t-test value; z: Mann Whitney U test.
